# Diabetic retinopathy: a comprehensive update on in vivo, in vitro and ex vivo experimental models

**DOI:** 10.1186/s12886-023-03155-1

**Published:** 2023-10-19

**Authors:** Muhammad Zulfiqah Sadikan, Nurul Alimah Abdul Nasir, Lidawani Lambuk, Rohimah Mohamud, Nur Hidayah Reshidan, Evon Low, Saiful Anuar Singar, Awis Sukarni Mohmad Sabere, Igor Iezhitsa, Renu Agarwal

**Affiliations:** 1 Department of Pharmacology, Faculty of Medicine, Manipal University College Malaysia (MUCM), Bukit Baru, 75150 Melaka, Malaysia; 2 Centre for Neuroscience Research (NeuRon), Faculty of Medicine, Universiti Teknologi MARA, 47000 Sungai Buloh, Selangor Malaysia; 3 Department of Immunology, School of Medical Sciences, Universiti Sains Malaysia, 16150 Kubang Kerian, Kelantan, Malaysia; 4 School of Biology, Faculty of Applied Sciences, Universiti Teknologi MARA, 40450 Shah Alam, Selangor Malaysia; 5 Ageing Biology Centre, Newcastle University, NE1 7RU Newcastle upon Tyne, UK; 6 Department of Nutrition and Integrative Physiology, College of Health and Human Sciences, Florida State University, 32306 Tallahassee, FL USA; 7 Kulliyyah of Pharmacy, International Islamic University Malaysia, Jalan Sultan Ahmad Shah, Bandar Indera Mahkota, 25200 Kuantan, Pahang, Malaysia; 8 School of Medicine, International Medical University, 57000 Bukit Jalil, Kuala Lumpur, Malaysia; 9 Department of Pharmacology and Bioinformatics, Volgograd State Medical University, Pavshikh Bortsov sq. 1, 400131 Volgograd, Russian Federation

**Keywords:** Diabetic retinopathy, Experimental models, In vitro, In vivo, Ex vivo

## Abstract

Diabetic retinopathy (DR), one of the leading causes of visual impairment and blindness worldwide, is one of the major microvascular complications in diabetes mellitus (DM). Globally, DR prevalence among DM patients is 25%, and 6% have vision-threatening problems among them. With the higher incidence of DM globally, more DR cases are expected to be seen in the future. In order to comprehend the pathophysiological mechanism of DR in humans and discover potential novel substances for the treatment of DR, investigations are typically conducted using various experimental models. Among the experimental models, in vivo models have contributed significantly to understanding DR pathogenesis. There are several types of in vivo models for DR research, which include chemical-induced, surgical-induced, diet-induced, and genetic models. Similarly, for the in vitro models, there are several cell types that are utilised in DR research, such as retinal endothelial cells, Müller cells, and glial cells. With the advancement of DR research, it is essential to have a comprehensive update on the various experimental models utilised to mimic DR environment. This review provides the update on the in vitro, in vivo, and ex vivo models used in DR research, focusing on their features, advantages, and limitations.

## Background

Diabetic retinopathy (DR) is a leading cause of visual defects and blindness, and the number of cases is increasing globally. Approximately 25% of diabetes mellitus (DM) patients have been diagnosed with DR [1]. In a recent meta-analysis by Teo et al. [2], the global prevalence of DR is approximately 22%, and that of vision-threatening DR is about 6%. The prevalence is higher among the Middle East, North Africa, and Western Pacific countries. With the increasing DM population worldwide, the global prevalence of DR is expected to rise from 103.2 million in 2022 to 160.5 million by 2045 [2].

202For effective management of DR, an in-depth understanding of the underlying molecular mechanisms is vital 202. The development of newer medical therapeutic options for DR requires an in-depth investigation into molecular and morphological changes and functional outcomes. The success of these investigations, to a great extent, is determined by the choice of appropriate experimental models. Experimental models refer to investigational subjects possessing artificially induced characteristics mimicking human conditions. These experimental models could comprise of animals that are subjected to induction of disease characteristics using various methods such as chemicals or surgical procedures. Some animals naturally develop diseases sufficiently similar to those found in humans and are used as experimental models.

Considering the importance of the experimental models of DR, the key characteristics of various in vitro, in vivo, and ex vivo models commonly used in DR research are reviewed and summarised in this work. The benefits and limitations of these models are also discussed to understand their context-specific utility. PubMed and Medline search engines were used to gather publications published between 1960 and 2021. This was accomplished using a single or combination of keywords, such as diabetic retinopathy, streptozotocin , in vivo, in vitro, and ex vivo. Only full-paper publications published in English were selected for this review.

## In vivo experimental models

### Chemical-induced DR

#### Background

The most common method of inducing diabetes pharmacologically is to use STZ, a naturally occurring antibiotic in *Streptomyces acromogenes*, or alloxan, a pyrimidine derivative [3]. STZ and alloxan destroy the pancreatic β-cells in the islets of Langerhans to exert their diabetogenic properties. There is a wide range selection of animals (e.g., rats, mice, rabbits, cats, dogs, pigs, non-human primates, and zebrafish) that have been studied to understand diabetes development through the administration of STZ or alloxan. Despite the splendid array of animal models, rodents are commonly used [3]. This could be due to their rapid breeding rate, small size, and short life span. Moreover, they are cost-effective. It must be noted that the type of animals selected is based on the objective of particular studies. Nevertheless, to date, no animal model has been able to reproduce a complete human DR in terms of vascular and neural complications in both the early and late progression of the disease [4].

#### Methods of induction

*STZ.* STZ is a glucosamine–nitrosourea compound targeting insulin-producing β-cells in the pancreas to mimic diabetogenic properties in humans [5]. There are more than a hundred analogues known. STZ occurs in nature as a 50–50 combination of (α and β anomers) [6]. The fundamental STZ structure has been kept in approximately one-third of the analogues, although alterations include acetylation, alkylation, and nitroso group substitution. Acetyl derivatives have the same activity as their parent molecule [6]. In an early report in 1963, STZ administration at 65 mg/kg was reported to induce type 1 DM (T1DM) in rats [7]. A higher dose of STZ is required for mice, which ranges from 150 to 400 mg/kg of STZ [5]. STZ administration also has been reported to induce type 2 DM (T2DM) through multiple low-dose injections, in combination with other chemicals (such as nicotinamide), or with dietary manipulations for the induction of diabetes in rodents and dogs [5–9]. In general, STZ-induced onset hyperglycaemia occurs within 48 h post-STZ administration regardless of the dosage and the hyperglycaemic condition can be maintained for up to 22–24 months [10]. STZ direct uptake to the pancreatic β-cell is aided by the glucose transporter 2 (GLUT2) receptor [11]. STZ executes its cytotoxic effects on the pancreatic islets of Langerhans through its nitrosourea moiety, where it causes DNA alkylation [3]. Other than that, DNA damage by STZ occurs due to increased production of reactive oxygen species (ROS) and nitric oxide (NO) [11]. STZ can be administered through intraperitoneal, intravenous, or subcutaneous injection resulting in prolonged hyperglycaemia and other characteristics of diabetes, such as polyuria and polydipsia [12].

Other than rodents, several animal species such as zebrafish, rabbits, dogs, pigs, beagles, and monkeys, are sensitive to the pancreatic β-cell cytotoxic effects of STZ [12–16]. Zebrafish were usually induced with multiple STZ doses, whereas huge animals such as rabbits, dogs, and nonhuman primates were usually induced with a single dose protocol. STZ-induced zebrafish model remained in a hyperglycaemic state around 80 days after induction of diabetes [15]. In contrast, huge animals may stay in a hyperglycaemic state for more than four years post-induction [16].

*Alloxan.* In 1942, before STZ was discovered, alloxan was employed to induce diabetes in animal models [17]. Alloxan is a uric acid derivative directly targeting pancreatic cells [18]. It was first created by Wöhler and Liebig by combining uric acid and nitric acid [19]. McLetchie and his colleagues discovered that intravenous injection of alloxan resulted in hypoglycaemia owing to necrosis in the pancreas islets of Langerhans while researching kidney problems in rabbits [17]. The death of cells resulted in the release of insulin reserves, resulting in hypoglycaemia, followed by the establishment of diabetes within 24 h. Similar to STZ, alloxan can also be administered via intraperitoneal and subcutaneous injection [19]. While rabbits induced with alloxan usually did not change their food intake frequency, rodents induced with alloxan usually have polydipsia, polyuria, glycosuria, and hyperglycaemia, all of which are common symptoms of diabetes [17]. The suppression of glucokinase, an enzyme implicated in the glucose-insulin regulation system and expressed in the liver and pancreas, is triggered by a direct cell death associated with alloxan. The compound can be toxic to liver and kidney cells, although toxicity can be prevented with careful dosage. Owing to the mode of action of alloxan being specific to pancreatic β-cells, the appropriate dosage is required to avoid possible toxicity accumulated in liver and kidney cells [20]. Nevertheless, a diabetic induce agent by alloxan is challenging to administer to animals due to its low stability in water at room and body temperature [17, 20]. All diabetic animal models administered with alloxan, including mice, rats, dogs, rabbits, and pigs, have experienced damage to their pancreatic β-cells and lead to DR due to retina-induced lesions [21–25].

*STZ vs. Alloxan*. Due to the higher ease of use and efficacy, STZ has become the gold standard agent over alloxan in inducing diabetes in vivo. This is due to more excellent stability of the STZ compound and extremely fast result in disease progression [26]. Conversely, alloxan is less favourable as it produces unpredictable and inconsistent results. Therefore, it could be more efficacious [27]. However, unsuccessful hyperglycaemia induction in experimental animals happens. It is associated with resistance to STZ or alloxan, which may be related to hormones or genetic indifference of different species or gender [27, 28].

#### Effects on retina

*STZ.* The induction of this chemical has been shown to induce DR in a wide range of animals, as mentioned above, and various dose regimens have been developed, depending on the type of animals. Nevertheless, rodents remain the most preferred animal model for the characterisation of the disease and therapeutic drug investigations. These animals are favoured even more than the non-human primates owing to their ability to develop hyperglycaemia one week after a single dose of STZ administration. They have been reported to exhibit various non-proliferative DR (NPDR) features.

In the STZ-induced mice model, the retinal morphologic alterations observed were thinning of the ganglion cell layer (GCL), inner plexiform layer (IPL), outer plexiform layer (OPL), and total retinal thickness as early as 3 to 4 weeks post-STZ induction [29]. Other than that, there were reduced retinal ganglion cells (RGCs) with increased astrocytes number, accompanied by reactive gliosis within 4 to 6 weeks post-STZ induction [30]. Meanwhile, at ten weeks post-STZ induction there was thinning of the inner nuclear layer (INL) and outer nuclear layer (ONL) [3]. For the microvascular changes, enhanced vascular permeability, pericyte loss, and microglial changes were reported at eight weeks [31, 32], neovascularisation (formation of new vessels) at 16 weeks [33], thickening of the capillary basal lamina at 17 weeks [34], and increased of acellular capillaries and pericyte ghosts within 6 to 9 months of STZ induction [10, 35, 36]. Continuous inflammation that occurs due to chronic hyperglycaemia leads to leukostasis (leukocytes plug within vessels) [35–38] and is associated with increased acellular capillary formation [35]. Hemodynamic and electroretinogram (ERG) instability were also reported within four weeks post-STZ induction including a decrease of arteriolar and venular flow velocity with decreased diameter and shear rates [39, 40], a reduced total oscillatory potential (OP) and OP3 amplitudes with prolonged of implicit times OP2-3 [41, 42]. In contrast to Kurihara et al. [41] and Sasaki et al. [42], the majority of studies failed to demonstrate the same declined trend for amplitudes of a-/b-wave, highlighting a conflicting outcome of mice as DR animal models [36]. Although multiple studies have recognised STZ as a diabetogenic chemical agent, researchers have yet to demonstrate consistent evidence on the effect of STZ diabetic-induced apoptotic and loss of RGC, thus remaining the subject of concern in recent times. It is worth noting that few studies demonstrated different numbers of RGC post-diabetic induction, such as an increased RGC apoptosis after two weeks [43] and decreased RGC numbers within 6 to 10 weeks after STZ administration [43, 44], supporting the notion of inconsistent results. Contrary to the aforementioned studies, others found no profound RGC apoptosis or GCL cell loss within ten months of post-hyperglycaemia [36, 38, 45]. These contrasting observations may be due to different strains of mice or regimens of STZ administration.

As opposed to mice, lower doses of STZ are required to induce diabetic in rats [5]. Several strains of rats were used to develop the DR model, including *Sprague Dawley* (SD), *Wistar*, and *Lewis*. Changes in the retinal morphology observed in rats were similar to those in mice. Thinning of the IPL and ONL were reported to be as early as four weeks post-STZ induction in SD rats [46, 47]. However, some studies also reported an increased total retinal layer thickness, which has been suggested to be due to retinal oedema [48, 49]. In fact, several authors reported retinal oedema as a retinal morphological feature of DR [50, 51]. Retinal oedema usually occurs after 12 to 16 weeks of STZ administration. *Wistar* and *Lewis* rats displayed degeneration of capillaries with the formation of pericyte ghosts within eight months of hyperglycaemia [52]. At the same time, *Lewis* and SD rats exhibit neuronal loss in GCL. Although the onset of retinal lesions varies with different rat strains, numerous phenotypes have been documented, including blood-retinal barrier (BRB) breakdown two weeks after diabetes onset [53], increased acellular capillaries containing degenerated intramural pericytes and apparent proliferation of endothelial cells after eight weeks [54], and thickening of basement membrane after one year [55]. Throughout the literature, studies have shown that DR development in rats has been linked to neuronal and glial damage before prominent vascular changes. It is identical to the mice model whereby progressive apoptosis and thinning of retinae were evident after 3 to 4 weeks of post-induction hyperglycaemia. Likewise, prominent gliosis with increased glial fibrillary acidic protein (GFAP) expression was observed at an early stage of four weeks of hyperglycaemia [56–58]. In addition, multiple studies also revealed a reduction of cells within GCL [52, 58 , 205] and astrocytes [53, 57] and an increase of microglia [52, 58] at 4 to 6 weeks of post-STZ induction. Subsequently, other vascular events include increased acellular capillaries [52, 59], leukostasis [60], thickening of capillary basement membrane [61], and pericyte loss [58, 61] were apparent at a more extended period (4 to 8 months) post-STZ induction. Other than the morphological changes, retinal dysfunction was also observed through reduced visual-behaviour responses [62], reduced a-/b-wave [56, 59, 63] and OP amplitude [56, 59] with delayed OP [63],increased retinal vessel diameter[203 , 204], retinal oxidative stress markers [64], retinal pro-inflammatory cytokines level [65] and retinal pro-angiogenic markers [64, 65].

Besides rodents, animals of different species have been examined with various results on disease development. For instance, 3 to 4 weeks post-STZ administration, zebrafish displayed thinning of IPL and photoreceptor segment layer, damaged cone receptor, and neuronal loss [15]. In contrast, rabbits induced with a single dosage of STZ acquired retinal and retinal haemorrhages, vascular lesions, venous thrombosis, and proliferative retinopathy after 4.5 months of induction [66]. STZ-induced DR dogs exhibit retinal lesions similar to those of human DR. In an extensive study of the DR dogs model, STZ administration caused a thickening capillary basement membrane (BM) after one year, pericyte and smooth muscle cell loss after four years, and microaneurysms, acellular capillaries, and intraretinal microvascular abnormalities (IRMAs) after seven years of hyperglycaemia [16]. Numerous studies have shown that loss of pericytes and microaneurysm formation depend on age, with younger animals exhibiting the features faster than older ones [67]. STZ-induced diabetic pigs displayed enhanced BRB permeability, INL, and GCL thinning and thickening capillary BM thickening [68, 69]. Accordingly, the tight junctions in the retina were weakened [68]. Monkeys induced with STZ remain diabetic for 6 to 15 years. However, no significant ocular changes were observed [70]. They usually developed hypertension and ischemic retinopathy with cotton-wool spots, and hyperfluorescent patches usually occur once they were hypertensive.

*Alloxan.* FOT_FB mice induced with alloxan acquired pericyte and RGCs loss post one week of induction, along with microaneurysms within three weeks post induction [29]. In another study using C57/Bl6 mice, alloxan induction caused microglial changes (thicker cell bodies and shorter dendrites) in the retina [31]. However, Gaucher et al. [31] failed to observe neuronal apoptosis, glial activation, microaneurysm, and haemorrhage formation, as demonstrated in comparable models. Nevertheless, they observed a significant reduction in the b-wave/a-wave amplitude ratio and an increment in the OP latency period in ERG after three months of alloxan induction [31].

Contrariwise to STZ, studies of alloxan-induced DR in rat models were limited. Neovascularisation and cataracts were observed between two to nine months and a year, respectively, post-alloxan administration [32, 71]. Pericyte ghosts, acellular capillaries, and thicker capillary BM were observed 15 months after alloxan induction [26, 71]. BRB breakdown, Müller glia growth, and endothelial enlargement were also reported with this induction model in rats [31, 72]. Five doses of alloxan within five weeks in dogs causes retinopathy comparable to DR in humans. However, dogs only acquired DR 53 to 69 months after the start of hyperglycaemia [22]. Alloxan-induced dogs developed haemorrhages, acellular capillaries, pericyte loss, and microaneurysms once DR appeared, making this a feasible model of PDR. Alloxan-induced pigs developed Müller cell contraction-promoting activity, which may be detected as early as 30 days following alloxan administration and can last up to 90 days [73]. These pigs developed cataracts [73], BRB breakdown, capillary collapse, and pericyte ghosts 60 days after receiving alloxan [44]. In contrast to dogs, the alloxan-induced pig model displayed several changes in NPDR.

The comparison of the chemical structure, mechanism of action, advantages, and disadvantages between STZ- and alloxan-induced models was described in Table 1.

**Table 1:** Comparison of the chemical structure, mechanism of action, advantages and disadvantages between STZ- and alloxan-induced model

	Streptozotocin(STZ)	Alloxan
Source	Glucosamine–nitrosourea compound derived from *Streptomyces achromogenes*	Synthesised from uric acid oxidation
Mechanism of Action	● Selective pancreatic β-cell uptake via GLUT2 ● Generates ROS ● Causes DNA fragmentation ● Acts as a NO donor ● Generates Adenosine triphosphate (ATP) de-phosphorylation ● β-cell necrotic death	● Selective pancreatic β-cell uptake via the GLUT2 ● Inhibit glucokinase ● Generates ROS ● β-cell necrotic death
Advantages	● Rapid simulation of natural T2DM disease progression ● Induced diabetes remains longer ● Cost-effective ● Easy handling (stable at 37 °C within one hour)	● High selective loss of pancreatic β-cell due to its inhibitory effect on glucokinase
Disadvantages	● Poor standardisation ● Carcinogenic to human	● Can be toxic to liver and kidney cells ● May cause spontaneous regeneration of β-cells ● Less stable in water at room and body temperature ● High variability ● High mortality

### Surgical-induced in vivo models

#### Background

One of the most extensively used methods to induce DR surgically in in vivo models is the removal of the pancreas, also known as pancreatectomy. Pancreatectomy can effectively mimic type 1 diabetes. Removing at least 90–95% of the pancreas can effectively induce hyperglycaemia in dogs [74]. Pancreatectomy was first discovered to increase blood sugar levels in dogs [75] following the invention of the complete pancreatectomy technique by Pfeiffer [76] in dogs. This technically challenging method has since been commonly used to create DR models, especially in larger animals, including cats, monkeys, and dogs.

#### Methods of induction

In cats’ models, pancreatectomy is usually performed with or without alloxan injection, which destroys pancreatic β-cells to induce T1DM [77]. In monkeys, the animals received insulin and oral pancreatic enzymes treatment after pancreatectomy. A detailed pancreatectomy method in the dog model has been fully described by Kumar et al. [78].

#### Effects on retina

*Cats.* Hyperglycaemia developed one to two weeks after pancreatectomy and was associated with early symptoms of DR, including thickening of the capillary BM as early as three months post-surgery [79]. Other than thickening of the capillary BM, microaneurysms were also acquired within five to six years post-surgery, followed by the development of retinal haemorrhages, capillary non-perfusion, and neovascularisation over the course of five to nine years [80]. Yet, two out of three long-term diabetic cats studied at seven years post-pancreatectomy developed only micro-aneurysms with no signs of haemorrhages or capillary non-perfusion. In contrast, the other cat showed no significant signs of DR [81].

*Monkeys.* Up to 94% of the alloxan-induced monkey models show no significant DR development within five years of hyperglycaemia after the surgery. It took 6 to 15 years post-surgery to develop mild blood-retinal barrier disruption [70]. Removal of the pancreas alone failed to induce DR in monkeys. However, spontaneous- or pharmacologically-induced hypertension in hyperglycaemic monkeys resulted in ischemic retinopathies [70]. Similar findings were seen in chemical-induced DR in monkeys. This suggests that retinal changes in monkeys depend on their hypertensive state, which may or may not correlate with the severity of the DR [82].

*Dogs.* Although dogs receiving pancreatectomy were used for DM research, there were no specific studies on the retina post pancreatectomy.

### Diet-induced in vivo models

#### Background

A high-fat or high-calorie diet is one of the main factors that exacerbate the progression of DM and its complications [83, 84]. Past studies have extensively utilised obese- or diabetic-induced rodents as models to understand the effects of these diets on the progression of DR. Other than these diets; the galactose diet was also commonly used to mimic hyperglycaemic changes in the retina [85].

#### Methods of induction

*High-fat diet (HFD).* HFD induction can be given alone or together with single or multiple low doses of STZ to induce insulin resistance, diabetes, and complication. There were two types of variations of HFD, (a) high-fat diet alone or (b) high-fat diet with a combination of high-sucrose or high-fructose diet (HSHF or HFHF). These methods of induction were mainly used in rodents. The HFD contains 40–65% kcal of fat compared to 4.5-10% kcal of fat in normal pellets [86–90]. For the variation with sucrose or fructose, 20–50% of sucrose or fructose were added to the diet [87, 88]. The initial age of HFD induction also varies among studies, where it ranges as early as five weeks old [91] to 10 weeks old [89]. Rodents were either induced with STZ at the start of HFD or after 2 to 8 weeks of HFD [86, 89, 90]. The impact of the percentage of fat in the diet, the presence of sucrose and fructose, the initial age of starting the diet, induction with STZ, and the duration of the diet will influence the findings on the animal models [92].

*Galactose.* Engerman and Kern [85] were the first to describe the animal model of DR induced by high-galactose diet. Animals such as mice, rats, rabbits, dogs, and zebrafish, have been described to have DR-like changes with a high-galactose diet [93]. The percentage of galactose used in the literature varies from 30 to 50% [94–96]. The initial age to start the galactose diet also varies from 4 weeks in rats [95] to 6 to 18 months old in dogs [25, 94]. The duration of diet also varies as short as seven months [97] to a long period of 41 months [98].

#### Effects on retina

*HFD.* The retinal lesion developed through HFD, with or without the addition of STZ induction, has been reviewed explicitly by Clarkson-Townsend et al. [88]. In summary, the retinal morphological changes in rodents receiving HFD were mixed. Several studies showed decreased retinal layer thickness and vascular density, increased acellular capillaries formation, vascular permeability, and vascular bleeding. In contrast, there were studies showing opposite retinal morphological changes or no changes at all. Other observed changes in retinal tissues include decreased a-/b- wave, delayed OP amplitude in ERG, and increased lipid peroxidases and pro-inflammatory cytokines.

*Galactose.* Retinal changes observed in mice on a high-galactose diet were endothelial cell loss and increased acellular capillaries at 15 months of hypergalactosaemia [99, 100], and pericyte loss, microaneurysms, and retinal thickening at 21 months of hypergalactosaemia [99, 101]. Other changes observed include the presence of pericyte ghosts and capillary BM thickening at 18 months [4] and gliosis by 28 months of hypergalactosaemia [102]. Galactose-fed animals, however, lack the metabolic irregularities experienced in chronic hyperglycaemia and advanced DR; hence this method is of relatively lesser interest [102].

### Genetic in vivo models

#### Background

In vivo, genetic models of DR have been used to investigate the pathogenesis and understand the treatment’s mechanism [103]. Spontaneous, strain-specific, and genetically edited mutations are among the genetic models that have been developed. Five identified DR mouse models include Ins2^Akita^ [104], non-obese diabetic (NOD) [102], db/db (Leprdb) [105], Akimba [106], and Kimba [107]. All of these mouse models varied in the mode of inheritance, disease aetiology, pathology, and disease progression [4, 103].

Another standard genetic model for DR is the rat model, which is slightly larger than mice but manageable at a lower cost. There are six established genetic DR rat models as follows; Zucker diabetic fatty (ZDF) [108], Otsuka Long-Evans Tokushima fatty (OLETF) [109], bio breeding (BB), WBN/Kob [110], spontaneously diabetic Torii (SDT) [111], and Goto-Kakizaki (GK) [112]. They are classified as monogenic and polygenic models according to the strains. The DR rat monogenic models are ZDF, OLETF, and BB, with independent mutations that perturb different nodes of the DR disease pathway [4]. Meanwhile, the DR rat polygenic models are SDT, WBN/Kob, and GK, which demonstrated the genetic complexity of DR [4].

#### Types of genetic models

*Ins2*^*Akita*^*mouse.* It is a standard model with a missense mutation in the Insulin 2 gene representing T1DM [104]. It has a dominant point mutation causing a conformational change in the insulin protein, resulting in protein accumulation in pancreatic β-cells and ultimately leading to β-cell death [113, 114]. The DR onset begins as early as four weeks of age [104].

*NOD mouse.* Another model for T1DM is the NOD mouse, where pancreatic β-cells are destroyed by CD4 + and CD8 + cells via autoimmune response [115, 116]. It is a polygenic model with several loci associated with the disease phenotype relatively similar in humans. NOD mice suffer from infiltration of dendritic cells and macrophages in pancreatic islets leading to inflammation, hyperglycaemia, and apoptosis of insulin-producing β-cells [117, 118]. The DR onset begins when spontaneous hyperglycaemia occurs in these mice at 12 weeks of age. A higher incidence of diabetes was observed in female compared to male rats, where at 30 weeks of age, around 80% of females and only 20% of males were reported to have diabetes [118].

*Lepr (db/db) mouse.* Alternatively, db/db (Lepr^db^) mice is an option for a DR study focusing on T2DM. It carries a mutation in the leptin receptor owing to leptin receptor deficiency which develops hyperglycaemia and obesity after four to eight weeks in homozygous mice [119, 120].

*Kimba mouse.* The Kimba mice is a transgenic nondiabetic model of proliferative retinopathy resulting from overexpression of vascular endothelial growth factor (VEGF) driven by the rhodopsin promoter in rhodopsin-expressing cells [121, 122]. The proliferative changes that usually occur at a later stage of DR developed early in this model. Therefore, the usage of this model limits the potential research in understanding the mechanism before the proliferative stage of DR.

*Akimba mouse.* The Akimba mouse is a model developed by cross-breeding Kimba mice with Ins2^Akita^ mice. This model inherits both the hyperglycaemic changes from the Ins2Akita model and the proliferative changes seen in the Kimba model [106, 123].

*ZDF rat.* A missense mutation in the leptin receptor gene, Lepr, causes insulin tolerance and excessive body weight gain in the ZDF DR rat model, which is adequate for severe spontaneous type 2 diabetes [108, 124]. The hyperglycaemia onset started at six weeks of age and continues throughout time.

*OLETF rat.* OLETF rat was created by selective breeding of Long-Evans rats that developed obesity, hyperglycaemia, and glycosuria [125]. It possesses a mutation in the G-protein coupled receptor GPR10’s initiation codon, which causes obesity and is ideal for mimicking T2DM [126]. The hyperglycaemia started at six months of age [127].

*BB rat.* BB rat is a monogenic DR model with a frameshift mutation in the immune-related nucleotide-binding protein gene Ian4, also known as *Ian5*, *Iddm1*, and *Gimap5*. This mutation causes the lymphopenia phenotype linked to auto-immune diabetes development [128–130].

*WBN/Kob rat.* The WBN/Kob rat is a spontaneous polygenic DR model, where the onset of hyperglycaemia appeared late, after 17 months old. This hyperglycaemia only affected male rats. Therefore, there might be the influence of sex hormones or chromosomal disorders. The diabetes appearance in this breed was suggested to be due to chronic pancreatitis [131]. Based on Mori et al. [132], chromosome 7, specifically the *Pdwk1* (pancreatitis and DM in WBN/Kob locus 1) region, was thought to be affected in this breed.

*SDT rats.* Another polygenic DR model specifically for nonobese T2DM is SDT rats. SDT rat is a substrain of SD rat [133]. The *Dmsdt1* was found to be the major gene locus responsible for pancreatic damage in this model [134]. The development of diabetes is more prominent in male rats, where glycosuria in male SDT rats appeared faster in the 20th week compared to females in the 45th week [135, 136].

*GK rats.* GK rat is a substrain of non-obese Wistar rat [137]. The mechanism behind the diabetes development of this model was suggested to be due to the anomaly of pancreatic IGF2 [138]. The development of diabetes is similar in male and female rats, where hyperglycaemia usually starts at four to six weeks of age [137]. The pancreas has an impaired capacity for β-cell neogenesis during the first four weeks of age, which is considered a pre-diabetic stage. The advantage of this breed is that diabetic cataracts appear late. Therefore, retinal microvasculature using fundus photography or OCT can be visualised easily [139].

#### Effects on retina

*Ins2*^*Akita*^*mouse.* The onset of DR appeared in the 8th week of hyperglycaemia indicated by increasing retinal vascular permeability and reactive microglia [140]. By the 12th week, abnormal swelling in somas, axons, and dendrites of RGCs was reported. As a result of inflammation, an increase in acellular capillaries and leukocytes in the vascular wall was observed after 36 weeks [104]. The reduction of IPL and INL thickness resulted in lower cholinergic and dopaminergic amacrine cell expression, as evidenced in the retina after six months of hyperglycaemia [104, 141]. However, part of the *rd8* mutation on *crb1* gene causes no retinal thinning or retinal architecture disruption at six months of age [103]. Previous studies indicated C57B1/6 N substrain (embryonic stem cells origin & mouse lines) contains the *crb1* gene in homozygous form; meanwhile, C57B1/6J substrain possesses the wild type of *crb1* gene [103]. The *rd8* mutation in C57B1/6 N substrain may influence the retinal degeneration findings. Therefore, researchers should look into this mutation when using this model [140].

*NOD mouse.* In the fourth week of hyperglycaemia, NOD mice showed apoptosis of pericytes, endothelial cells, and RGCs with retinal capillary BM thickening [142]. In comparison, Azrad-Leibovich et al. [102] reported the presence of retinal oedema with the absence of microaneurysm capillary leakage as early as two weeks after hyperglycaemia onset. However, in another study by Zorrilla-Zubilete et al. [143], retinal oedema was absent at two weeks of hyperglycaemia onset. Reduced arteriole diameter was reported at three weeks of hyperglycaemia onset [144]. Similarly, Shaw et al. [145] reported vasoconstriction, degeneration of major vessels with abnormal microvessels, and disordered focal proliferation of vessels four months after hyperglycaemia onset. Other than morphology and vascular changes, increased retinal angiogenic VEGF level was also reported with this model [142].

*Lepr (db/db) mouse.* Reduction in RGCs number and increase in the total central retina, INL, and photoreceptor layer thickness were identified after six weeks of hyperglycaemia [146]. Gliosis reactivation and pericyte loss were reported at 4.5 to 13 months from the onset of hyperglycaemia [146]. There were also increased BRB breakdown, retinal capillary density, retinal angiogenic VEGF level, oxidative stress markers, and pro-inflammatory cytokines observed at 5 to 13 months of hyperglycaemia onset [147, 148]. The ERG findings showed a reduction of a-, b-, and c-waves at 24 weeks old [149]. Samuels et al. [150] observed that the reduction of the b-wave preceded the reduction of the a-wave at 16 weeks old.

*Kimba and Akimba mice.* The INL and ONL were reduced in Kimba mice at seven days postnatal [121]. By 28 days postnatal, microvascular abnormalities and capillary dropout were observed and continued until nine weeks of age, where pericyte loss was detected [151, 152]. The pericyte loss and retinal neovascularisation with diffuse vascular leakage were found in the late-stage DR, which were well-characterised by this breed [106, 153]. The Akimba mouse has leaky capillaries, tortuous vessels, and microaneurysms in the retina by 8 weeks old [106]. With disease progression, increased photoreceptor loss, decreased retina thickness, increased oedema persistence, and retinal detachment were observed [106].

*ZDF rat.* The capillary BM thickening and cell nuclear density increased at 6 to 7 months of age [154], whereas increased apoptotic capillary cells were observed after 20 weeks of age [155]. Szabó et al., 2017 [156] observed retinal oedema and microglial activation at 32 weeks of age with no retinal cell apoptosis. At an early age (6 to 7 months old), the absence of acellular capillaries and pericyte loss were reported, but both conditions then appeared at a later stage [157–159]. Lowering of b-wave amplitude in ERG was seen as early as 12 weeks of age [155].

*OLETF rat.* Microvessel-related symptoms appeared six weeks after hyperglycaemia onset, including leukocyte entrapment in the retinal microcirculation [125]. The reduction in pericytes and damage of endothelial cells were detected at three months post hyperglycaemia [131]. The increased thickness of the capillary BM, microaneurysms, capillary formation in loops, and tortuosity were also detected [125, 160]. The ERG findings of OLETF rats were prolonged latency of OP amplitude at 12.5 months old [161]. However, no acellular capillaries formation and pericyte ghosts were observed in this model [162].

*BB rat.* BB rats acquired retinal lesions such as pericyte loss, capillary degeneration, and microaneurysms by eight to eleven months old [163–165]. Meanwhile, Blair et al. [166] reported increased BRB breakdown and abnormal retinal microvasculature at eight months of age.

*WBN/Kob rat*. This breed acquired late-onset hyperglycaemia (9 to 12 months of age). Therefore, capillary BM thickening [167] and neovascularisation [168, 169] also appeared later at 14 to 24 months old. However, retinal degeneration, such as loss of rods and cones (outer retinal layer), appeared early before hyperglycaemia onset [167, 170].

*SDT rat.* Retinal detachment with fibrous proliferation, retinal ischemia with neovascularisation, leukostasis, increased number of apoptotic cells in the GCL and INL, pericyte loss, and vascular lesions were reported in aged SDT rats (after 12 months of age) [133, 171–173]. A distinguishing feature similar to retinal detachment observed in humans was the formation of massive retinal folds with significant leakage around the optic disc [133, 171–173]. However, the absence of microaneurysms makes them a more suitable model for studying NPDR.

*GK rat.* An increase in BRB permeability was reported by three months old, followed by an increase in the endothelial/pericyte ratio at seven months old [139, 174]. Retinal microvascular cells apoptosis, higher acellular capillary formation, and pericyte loss occurred even in mild hyperglycaemic GK rats at one year old [175]. Additionally, retinal layer thickening and reduced a- and b-waves amplitude in ERG were reported at 12 months old [176]. Retinal angiogenic markers (VEGF and matrix metalloproteinase (MMPs)) were noted to be increased by seven months old of age and not earlier than that [177, 178].

## In vitro model

Since pathological retinal angiogenesis is the main reason for progression in DR, most in vitro cell culture utilises isolated endothelial cells to mimic human angiogenic processes [179]. The most commonly used retinal endothelial cells are bovine retinal endothelial cells (BRECs) [180], human retinal endothelial cells (HRECs) [181], and Rhesus monkey retinal and choroid endothelial cells (RF/6a) [182]. However, some studies also utilised non-retinal specific endothelial cells and focused on the angiogenesis process. The non-retinal specific endothelial cells used include human umbilical vein endothelial cells (HUVECs) [183], human microvascular endothelial cells (HMVECs) [184], and human dermal microvascular endothelial cells (HMVEC-D) [185]. The cells were treated with high glucose concentrations, imitating a diabetic condition. The target mechanism in in vitro studies using endothelial cells is an angiogenic process. Therefore, the commonly assessed markers include growth factors such as VEGF, extracellular matrix proteins such as MMPs, and signalling pathways involving oxidative stress, inflammation, and apoptosis.

Other cell types used in the in vitro model were specific retinal cells, such as pericytes, fibroblasts, macrophages, and Müller or other glial cells [186]. Glial cells are important neuronal tissue supporters, which assist communication between vessels and neurons. Studies using glial cells usually explore the relationship between vasculopathy and neuropathy in DR [53]. Among all the glial cells, Müller cells were commonly used in studying DR [187]. The disadvantage of in vitro culture is the absence of a retinal microenvironment that can be found in vivo model [188].

## Ex vivo models

For ex vivo models of DR, studies usually used organotypic retinal explant cultures to mimic the structure of the tissues nearer to in vivo environment, overtaking some of the constraints of the in vitro studies [189]. Most studies using retinal explant models did research on assessing the toxicity of potential new chemicals or drugs [190] or evaluating retina functionality [191]. The initial concept of using retinal explants to investigate proliferative retinopathy was initiated by Forrester et al. [192], where bovine retinal explants cultured in collagen gels were used. Currently, the use of collagen was replaced by synthetic Matrigel, which improved vessel sprouting [189] and provided consistent results. This replacement was also recommended by the international consensus [193]. Other than bovine, mice retinal explant cultures were commonly used to study neovascularisation [194]. Vitreoretinal angiogenesis progression can also be identified using time-sequential imaging in an ex vivo model [195]. Until present, retinal explants are normally cultured with stimuli that portray the retinal environment in DR, such as high glucose, oxidative stress, or advanced glycation end-products (AGE) [196]. Other than that, the retina from the in vivo DR model was used for the ex vivo in vitro culture once the retinal neovascularisation development had taken place to represent the PDR process [197–200]. The use of retinal explants still needs to be improved in research, possibly due to the expertise requirements to isolate retinas and their unsuitability for long-term experimental periods [201].

## Conclusions and perspectives

Experimental models of DR are used to study the pathophysiology, mechanisms, and potential treatments for the disease. These models aim to replicate the key features and progression of DR observed in humans. Among all the experimental models, in vivo model is most preferable due to several limitations of in vitro and ex vivo models, such as the absence of a retinal microenvironment and complex retina isolation, respectively. The in vivo model has the most significant advantage, as it can closely mimic DR-like human conditions. Among in vivo models, chemically induced diabetes by STZ is the most commonly used in vivo model. Although experimental models can be used to predict disease mechanisms or in potential treatment, they may not accurately be translated to human conditions as they do not fully replicate the complexity and heterogeneity of the disease seen in humans. However, the use of specific experimental models may target particular mechanism which is limited in human due to the limitation of the sample. Therefore, the researchers must choose an appropriate experimental DR model to address their research objectives.

Following findings from experimental models, clinical studies involving human subjects, such as clinical trials and observational studies, is vital to provide essential evidence that considers the genetic and environmental factors that influence disease pathophysiology, progression, and response to therapies. Clinical studies also ensure that the findings from experimental models are rigorously evaluated and their clinical implications are thoroughly understood. In summary, while experimental models of DR provide crucial insights into the underlying mechanisms and aid in developing treatment strategies, clinical validation is necessary to ensure the findings can be translated into meaningful outcomes for patients.

## Data Availability

Not applicable..

## List of abbreviations

**AGE**: Advanced glycation end-products
**ATP**: Adenosine triphosphate
**BB**: Biobreeding
**BRB**: Blood-retinal barrier
**BRECs**: Bovine retinal endothelial cells
**DM**: Diabetes mellitus
**DNA**: Deoxyribonucleic acid
**DR**: Diabetic retinopathy
**ERG**: Electroretinogram
**GCL**: Ganglion cell layer
**GFAT2**: glutamine-fructose-6-phosphate amidotransferase
**GK**: Goto-Kakizaki
**GLUT2**: Glucose transporter 2
**HFD**: High-fat diet
**HFHF**: high-fat/high-fructose
**HMVEC-D**: Human dermal microvascular endothelial cells
**HMVECs**: Human microvascular endothelial cells
**HRECs**: Human retinal endothelial cells
**HUVECs**: Human umbilical vein endothelial cells
**INL**: Inner nuclear layer
**IPL**: Inner plexiform layer
**IRMAs**: Intraretinal microvascular abnormalities
**NO**: Nitric oxide
**NOD**: Non-obese diabetic
**NR4A1**: Nuclear receptor 4A1
**OLETF**: Otsuka Long-Evans Tokushima fatty
**ONL**: Outer nuclear layer
**OP**: Oscillatory potential
**OPL**: Outer plexiform layer
**PDR/NPDR**: Proliferative/Non-proliferative diabetic retinopathy
**Rac1**: Ras-related C3 botulinum toxin substrate 1
**RF/6a**: Rhesus monkey retinal and choroid endothelial cells
**ROS**: Reactive oxygen species
**SDT**: Spontaneously diabetic Torii
**STZ**: Streptozotocin
**T1DM/T2DM**: Type 1/Type 2 diabetes mellitus
**ZDF**: Zucker diabetic fatty

## References

[1] Saeedi P, Petersohn I, Salpea P, Malanda B, Karuranga S, Unwin N, Colagiuri S, Guariguata L, Motala AA, Ogurtsova K (2019). Global and regional diabetes prevalence estimates for 2019 and projections for 2030 and 2045: results from the International Diabetes Federation Diabetes Atlas. Diabetes Res Clin Pract.

[2] Teo ZL, Tham Y-C, Yu MCY, Chee ML, Rim TH, Cheung N, Bikbov MM, Wang YX, Tang Y, Lu Y (2021). Global prevalence of Diabetic Retinopathy and Projection of Burden through 2045: systematic review and Meta-analysis. Ophthalmology.

[3] Rakieten N, Rakieten ML, Nadkarni V (1963). Studies on the diabetogenic action of streptozotocin (NSC-37917). Cancer Chemother Rep.

[4] Olivares AM, Althoff K, Chen GF, Wu S, Morrisson MA, DeAngelis MM, Haider N (2017). Animal models of diabetic retinopathy. Curr Diab Rep.

[5] Furman BL (2015). Streptozotocin-induced diabetic models in mice and rats. Curr Protoc Pharmacol.

[6] Agarwal M (1980). Streptozotocin: mechanisms of action. FEBS Lett.

[7] Junod A, Lambert A, Orci L, Pictet R, Gonet A, Renold A (1967). Studies of the diabetogenic action of streptozotocin. Proc Soc Exp Biol Med.

[8] Gajdosik A, Gajdosikova A, Stefek M, Navarova J, Hozova R (1999). Streptozotocin-induced experimental diabetes in male Wistar rats. Gen Physiol Biophys.

[9] Ghasemi A, Khalifi S, Jedi S. Streptozotocin-nicotinamide-induced rat model of type 2 diabetes. Acta Physiol Hung *2014*, 101(4):408–20. 10.1556/APhysiol.101.2014.4.2 25532953

[10] Feit-Leichman RA, Kinouchi R, Takeda M, Fan Z, Mohr S, Kern TS, Chen DF. Vascular damage in a mouse model of diabetic retinopathy: relation to neuronal and glial changes. Invest Ophthalmol Vis Sci 2005, 46(11):4281–7. 10.1167/iovs.04-1361 16249509

[11] Eleazu CO, Eleazu KC, Chukwuma S, Essien UN (2013). Review of the mechanism of cell death resulting from streptozotocin challenge in experimental animals, its practical use and potential risk to humans. J Diabetes Metab Disord.

[12] Vieira R, Souto SB, Sánchez-López E, López Machado A, Severino P, Jose S, Santini A, Silva AM, Fortuna A, García ML. Sugar-lowering drugs for type 2 diabetes mellitus and metabolic syndrome—strategies for in vivo administration: part-II. J Clin Med 2019, 8(9):1332. 10.3390/jcm8091332 PMC6780268 31466386

[13] Vique-Sánchez JL, López-Palacios TP, Miranda-Ozuna JF, Benítez-Cardoza CG. Effects of W100E-Leptin in streptozotocin-induced diabetic mice. Nutr Clin y Diet Hosp 2020, 40(3). 10.12873/403vique.

[14] Jin D, Zhang B, Li Q, Tu J, Zhou B. Effect of punicalagin on multiple targets in streptozotocin/high-fat diet-induced diabetic mice. Food Funct 2020, 11(12):10617–34. 10.1039/d0fo01275k 33210684

[15] Intine RV, Olsen AS, Sarras MP (2013). A zebrafish model of diabetes mellitus and metabolic memory. J Vis Exp.

[16] Gardiner T, Stitt A, Anderson H, Archer D (1994). Selective loss of vascular smooth muscle cells in the retinal microcirculation of diabetic dogs. Br J Ophthalmol.

[17] McLetchie N (2002). Alloxan diabetes: a discovery, albeit a minor one. J R Coll Physicians Edinb.

[18] Dixon KC, King A, Malinin T. Protein in dying β-cells of the pancreatic islets. Q J Exp Physiol Cogn Med Sci *1960*, 45(2):202–12. 10.1113/expphysiol.1960.sp001458 13816993

[19] Rohilla A, Ali S. Alloxan induced diabetes: mechanisms and effects. Int J Res Pharm Biomed Sci 2012, 3(2):819–23.

[20] Borg L (1981). Effects of alloxan on the islets of Langerhans: why does alloxan not stimulate insulin release?. Ups J Med Sci.

[21] de Roetth A Jr, Pei YF. Experimental diabetic retinopathy: retinal metabolism in the alloxan diabetic rat. Arch Ophthalmol 1960, 63(2):226–31. 10.1001/archopht.1960.00950020228004 13814991

[22] Engerman R, Bloodworth J (1965). Experimental diabetic retinopathy in dogs. Arch Ophthalmol.

[23] Kern TS, Engerman RL (1994). Comparison of retinal lesions in alloxan-diabetic rats and galactose-fed rats. Curr Eye Res.

[24] Radenković M, Stojanović M, Prostran M. Experimental diabetes induced by alloxan and streptozotocin: the current state of the art. J Pharmacol Toxicol Methods *2016*, 78:13–31. 10.1016/j.vascn.2015.11.004 26596652

[25] Zarebska A, Czerny K, Bakiera K, Cichacz-Kwiatkowska B, Lis-Sochocka M, Kiś G, Wojtowicz Z. Histological changes in the retina in experimental alloxan-induced diabetes in rabbits. In: Ann Univ Mariae Curie Sklodowska Med: 2001; 2001:81–84. 11977369

[26] Szkudelski T. The mechanism of alloxan and streptozotocin action in B cells of the rat pancreas. Physiol Res 2001, 50(6):537–46. 11829314

[27] Misra M, Aiman U (2012). Alloxan: an unpredictable drug for diabetes induction?. Indian J Pharmacol.

[28] Lenzen S. The mechanisms of alloxan-and streptozotocin-induced diabetes. Diabetologia 2008, 51(2):216–26. 10.1007/s00125-007-0886-7 18087688

[29] Weerasekera LY, Balmer LA, Ram R, Morahan G. Characterization of retinal vascular and neural damage in a novel model of diabetic retinopathy. Invest Ophthalmol Vis Sci 2015, 56(6):3721–30. 10.1167/iovs.14-16289 26047174

[30] Kumar S, Zhuo L (2010). Longitudinal in vivo imaging of retinal gliosis in a diabetic mouse model. Exp Eye Res.

[31] Gaucher D, Chiappore J-A, Pâques M, Simonutti M, Boitard C, Sahel JA, Massin P, Picaud S. Microglial changes occur without neural cell death in diabetic retinopathy. Vis Res 2007, 47(5):612–23. 10.1016/j.visres.2006.11.017 17267004

[32] Schröder S, Palinski W, Schmid-Schönbein G. Activated monocytes and granulocytes, capillary nonperfusion, and neovascularization in diabetic retinopathy. Am J Pathol *1991*, 139(1):81–100. PMC1886150 1713023

[33] Su L, Ji J, Bian J, Fu Y, Ge Y, Yuan Z (2012). Tacrolimus (FK506) prevents early retinal neovascularization in streptozotocin-induced diabetic mice. Int Immunopharmacol.

[34] Kuiper EJ, Zijderveld Rv, Roestenberg P, Lyons KM, Goldschmeding R, Klaassen I, Noorden CJV, Schlingemann RO (2008). Connective tissue growth factor is necessary for retinal capillary basal lamina thickening in diabetic mice. J Histochem Cytochem.

[35] Gubitosi-Klug RA, Talahalli R, Du Y, Nadler JL, Kern TS. 5-Lipoxygenase, but not 12/15-lipoxygenase, contributes to degeneration of retinal capillaries in a mouse model of diabetic retinopathy. Diabetes 2008, 57(5):1387–93. 10.2337/db07-1217 PMC4444435 18346986

[36] Zheng L, Du Y, Miller C, Gubitosi-Klug R, Kern T, Ball S, Berkowitz B. Critical role of inducible nitric oxide synthase in degeneration of retinal capillaries in mice with streptozotocin-induced diabetes. Diabetologia 2007, 50(9):1987–96. 10.1007/s00125-007-0734-9 17583794

[37] Kubota S, Ozawa Y, Kurihara T, Sasaki M, Yuki K, Miyake S, Noda K, Ishida S, Tsubota K. Roles of AMP-activated protein kinase in diabetes-induced retinal inflammation. Invest Ophthalmol Vis Sci *2011*, 52(12):9142–8. 10.1167/iovs.11-8041 22058332

[38] Li G, Tang J, Du Y, Lee CA, Kern TS. Beneficial effects of a novel RAGE inhibitor on early diabetic retinopathy and tactile allodynia. Mol Vis 2011, 17:3156–65. PMC3235538 22171162

[39] Wang Z, Yadav AS, Leskova W, Harris NR (2010). Attenuation of streptozotocin-induced microvascular changes in the mouse retina with the endothelin receptor A antagonist atrasentan. Exp Eye Res.

[40] Wright WS, Harris NR (2008). Ozagrel attenuates early streptozotocin-induced constriction of arterioles in the mouse retina. Exp Eye Res.

[41] Kurihara T, Ozawa Y, Nagai N, Shinoda K, Noda K, Imamura Y, Tsubota K, Okano H, Oike Y, Ishida S. Angiotensin II type 1 receptor signaling contributes to synaptophysin degradation and neuronal dysfunction in the diabetic retina. Diabetes 2008, 57(8):2191–8. 10.2337/db07-1281 PMC2494692 18487452

[42] Sasaki M, Ozawa Y, Kurihara T, Kubota S, Yuki K, Noda K, Kobayashi S, Ishida S, Tsubota K (2010). Neurodegenerative influence of oxidative stress in the retina of a murine model of diabetes. Diabetologia.

[43] Martin PM, Roon P, Van Ells TK, Ganapathy V, Smith SB. Death of retinal neurons in streptozotocin-induced diabetic mice. Invest Ophthalmol Vis Sci 2004, 45(9):3330–6. 10.1167/iovs.04-0247 15326158

[44] Yang Y, Hayden MR, Sowers S, Bagree SV, Sowers JR (2010). Retinal redox stress and remodeling in cardiometabolic syndrome and diabetes. Oxid Med Cell Longev.

[45] Howell SJ, Mekhail MN, Azem R, Ward NL, Kern TS. Degeneration of retinal ganglion cells in diabetic dogs and mice: relationship to glycemic control and retinal capillary degeneration. Mol Vis 2013, 19:1413. PMC3695756 23825921

[46] Aizu Y, Oyanagi K, Hu J, Nakagawa H (2002). Degeneration of retinal neuronal processes and pigment epithelium in the early stage of the streptozotocin-diabetic rats. Neuropathol.

[47] Zhang J, Wu Y, Jin Y, Ji F, Sinclair SH, Luo Y, Xu G, Lu L, Dai W, Yanoff M (2008). Intravitreal injection of erythropoietin protects both retinal vascular and neuronal cells in early diabetes. Invest Ophthalmol Vis Sci.

[48] Deguchi S, Otake H, Nakazawa Y, Hiramatsu N, Yamamoto N, Nagai N (2017). Ophthalmic formulation containing nilvadipine nanoparticles prevents retinal dysfunction in rats injected with streptozotocin. Int J Mol Sci.

[49] Qiu F, Zhu M, Le YZ (2019). Noninvasive diagnosis of regional alteration of retinal morphology and structure with optical coherence tomography in rodents. Adv Exp Med Biol.

[50] Lu LC, Zhou W, Li ZH, Yu CP, Li CW, LuoH-h, Xie H (2012). Effects of arctiin on streptozotocin-induced diabetic retinopathy in Sprague-Dawley rats. Planta Med.

[51] Yu X, Xu Z, Mi M, Xu H, Zhu J, Wei N, Chen K, Zhang Q, Zeng K, Wang J (2008). Dietary taurine supplementation ameliorates diabetic retinopathy via anti-excitotoxicity of glutamate in streptozotocin-induced Sprague-Dawley rats. Neurochem Res.

[52] Kern T, Miller C, Tang J, Du Y, Ball S, Berti-Matera L (2010). Comparison of three strains of diabetic rats with respect to the rate at which retinopathy and tactile allodynia develop. Mol Vis.

[53] Rungger–Brändle E, Dosso AA, Leuenberger PM (2000). Glial reactivity, an early feature of diabetic retinopathy. Invest Ophthalmol Vis Sci.

[54] Li L, Li YL, Zhou YF, Ge ZY, Wang LL, Li ZQ, Guo YJ, Jin L, Ren Y, Liu JX (2017). Jiangtang Xiaozhi recipe prevents diabetic retinopathy in streptozotocin-induced diabetic rats. Chin J Integr Med.

[55] Anderson H, Stitt A, Gardiner T, Lloyd S, Archer D (1993). Induction of alloxan/streptozotocin diabetes in dogs: a revised experimental technique. Lab Anim.

[56] Li Q, Zemel E, Miller B, Perlman I (2002). Early retinal damage in experimental diabetes: electroretinographical and morphological observations. Exp Eye Res.

[57] Ly A, Yee P, Vessey KA, Phipps JA, Jobling AI, Fletcher EL (2011). Early inner retinal astrocyte dysfunction during diabetes and development of hypoxia, retinal stress, and neuronal functional loss. Invest Ophthalmol Vis Sci.

[58] Zeng XX, NG YK, Ling EA (2000). Neuronal and microglial response in the retina of streptozotocin-induced diabetic rats. Vis Neurosci.

[59] Si YF, Wang J, Guan J, Zhou L, Sheng Y, Zhao J (2013). Treatment with hydrogen sulfide alleviates streptozotocin-induced diabetic retinopathy in rats. Br J Pharmacol.

[60] Jariyapongskul A, Rungjaroen T, Kasetsuwan N, Patumraj S, Seki J, Niimi H (2007). Long-term effects of oral vitamin C supplementation on the endothelial dysfunction in the iris microvessels of diabetic rats. Microvasc Res.

[61] Anderson H, Stitt A, Gardiner T, Archer D (1995). Diabetic retinopathy: morphometric analysis of basement membrane thickening of capillaries in different retinal layers within arterial and venous environments. Br J Ophthalmol.

[62] Sadikan MZ, Abdul Nasir NA, Iezhitsa I, Agarwal R (2021). Open field mirror test as a tool for the assessment of visual functions in rats with streptozotocin-induced diabetes. Neurosci Res Notes.

[63] Hancock HA, Kraft TW (2004). Oscillatory potential analysis and ERGs of normal and diabetic rats. Invest Ophthalmol Vis Sci.

[64] Sadikan MZ, Abdul Nasir NA, Iezhitsa I, Agarwal R. Antioxidant and anti-apoptotic effects of tocotrienol-rich fraction against streptozotocin-induced diabetic retinopathy in rats. Biomed Pharmacother 2022, *153*:113533. 10.1016/j.biopha.2022.113533. 10.1016/j.biopha.2022.113533 36076612

[65] Sadikan MZ, Abdul Nasir NA, Bakar NS, Iezhitsa I, Agarwal R. Tocotrienol-rich fraction reduces retinal inflammation and angiogenesis in rats with streptozotocin-induced diabetes. BMC Complement Med Ther 2023 Jun 2;23(1):179. 10.1186/s12906-023-04005-9. 10.1186/s12906-023-04005-9 PMC10236897 37268913

[66] Drago F, La Manna C, Emmi I, Marino A (1998). Effects of sulfinpyrazone on retinal damage induced by experimental diabetes mellitus in rabbits. Pharmacol Res.

[67] Kador PF, Takahashi Y, Akagi Y, Blessing K, Randazzo J, Wyman M (2007). Age-dependent retinal capillary pericyte degeneration in galactose-fed dogs. J Ocul Pharmacol Ther.

[68] Lee SE, Ma W, Rattigan EM, Aleshin A, Chen L, Johnson LL, D’Agati VD, Schmidt AM, Barile GR (2010). Ultrastructural features of retinal capillary basement membrane thickening in diabetic swine. Ultrastruct Pathol.

[69] Hainsworth DP, Katz ML, Sanders DA, Sanders DN, Wright EJ, Sturek M (2002). Retinal capillary basement membrane thickening in a porcine model of diabetes mellitus. Comp Med.

[70] Tso M, Kurosawa A, Benhamou E, Bauman A, Jeffrey J, Jonasson O (1988). Microangiopathic retinopathy in experimental diabetic monkeys. Trans Am Ophthalmol Soc.

[71] Spadella CT, Machado JLM, Lerco MM, Ortolan E, Schellini SA, Gregório E. Temporal relationship between successful pancreas transplantation and control of ocular complications in alloxan-induced diabetic rats. In: *Transplant Proc: 2008*: Elsevier; 2008: 518–523. 10.1016/j.transproceed.2008.01.049 18374119

[72] Doczi-Keresztesi Z, Jung J, Kiss I, Mezei T, Szabo L, Ember I (2012). Retinal and renal vascular permeability changes caused by stem cell stimulation in alloxan-induced diabetic rats, measured by extravasation of fluorescein. In Vivo.

[73] King JL, Mason JO, Cartner SC, Guidry C (2011). The influence of alloxan-induced diabetes on Müller cell contraction-promoting activities in vitreous. Invest Ophthalmol Vis Sci.

[74] Rastellini C, Shapiro R, Corry R, Fung J, Starzl T, Rao A. An attempt to reverse diabetes by delayed islet cell transplantation in humans. In: Transplant Proc: 1997: NIH Public Access; 1997: 2238. 10.1016/s0041-1345(97)00313-8 PMC2966316 9193607

[75] Banting F, Best C. Pancreatic extracts. 1922. J Lab Clin Med. 1990, 115(2):254–272. 2405086

[76] Pfeiffer EF. Handbook of diabetes mellitus. Volume 1. JF Lehmanns Verlag; 1969.

[77] Maqbool M, Dar MA, Gani I, Mir SA (2019). Animal models in diabetes mellitus: an overview. J Drug Deliv Ther.

[78] Kumar S, Singh R, Vasudeva N, Sharma S (2012). Acute and chronic animal models for the evaluation of anti-diabetic agents. Cardiovasc Diabetol.

[79] Mansour S, Hatchell D, Chandler D, Saloupis P, Hatchell M. Reduction of basement membrane thickening in diabetic cat retina by sulindac. Invest Ophthalmol Vis Sci 1990, 31(3):457–63. 2318584

[80] Hatchell DL (1995). Diabetic retinopathy in a cat. Exp Eye Res.

[81] Linsenmeier RA, Braun RD, McRipley MA, Padnick LB, Ahmed J, Hatchell D, McLeod DS, Lutty GA (1998). Retinal hypoxia in long-term diabetic cats. Invest Ophthalmol Vis Sci.

[82] Kim SY, Johnson MA, McLeod DS, Alexander T, Otsuji T, Steidl SM, Hansen BC, Lutty GA (2004). Retinopathy in monkeys with spontaneous type 2 diabetes. Invest Ophthalmol Vis Sci.

[83] Beigrezaei S, Ghiasvand R, Feizi A, Iraj B (2019). Relationship between dietary patterns and incidence of type 2 diabetes. Int J Prev Med.

[84] Sami W, Ansari T, Butt NS, Ab Hamid MR (2017). Effect of diet on type 2 diabetes mellitus: a review. Int J Health Sci (Qassim).

[85] Engerman RL, Kern TS (1984). Experimental galactosemia produces diabetic-like retinopathy. Diabetes.

[86] Chang RC, Shi L, Huang CC, Kim AJ, Ko ML, Zhou B, Ko GY (2015). High-fat diet–induced retinal dysfunction. Invest Ophthalmol Vis Sci.

[87] Asare-Bediako B, Noothi SK, Li Calzi S, Athmanathan B, Vieira CP, Adu-Agyeiwaah Y, Dupont M, Jones BA, Wang XX, Chakraborty D, Levi M (2020). Characterizing the retinal phenotype in the high-fat diet and western diet mouse models of prediabetes. Cells.

[88] Clarkson-Townsend DA, Douglass AJ, Singh A, Allen RS, Uwaifo IN, Pardue MT. Impacts of high fat diet on ocular outcomes in rodent models of visual disease. Exp Eye Res 2021, 204:108440. 10.1016/j.exer.2021.108440. 10.1016/j.exer.2021.108440 PMC7946735 33444582

[89] Kowluru RA. Retinopathy in a diet-induced type 2 diabetic rat model and role of epigenetic modifications. Diabetes 2020, 69(4):689–98. 10.2337/db19-1009 PMC7085254 31949005

[90] Barrière DA, Noll C, Roussy G, Lizotte F, Kessai A, Kirby K, Belleville K, Beaudet N, Longpré JM, Carpentier AC, Geraldes P (2018). Combination of high-fat/high-fructose diet and low-dose streptozotocin to model long-term type-2 diabetes complications. Sci Rep.

[91] Zhang Q, Xiao X, Zheng J, Li M, Yu M, Ping F, Wang T, Wang X (2018). Compound danshen dripping pill inhibits retina cell apoptosis in diabetic rats. Front Physiol.

[92] Skovsø S (2014). Modeling type 2 diabetes in rats using high fat diet and streptozotocin. J Diabetes Investig.

[93] Jo DH, Cho CS, Kim JH, Jun HO, Kim JH (2013). Animal models of diabetic retinopathy: doors to investigate pathogenesis and potential therapeutics. J Biomed Sci.

[94] Kador PF, Akagi Y, Takahashi Y, Ikebe H, Wyman M, Kinoshita JH (1990). Prevention of retinal vessel changes associated with diabetic retinopathy in galactose-fed dogs by aldose reductase inhibitors. Arch Ophthalmol.

[95] Robinson WG, Laver NM, Lou MF (1995). The role of aldose reductase in diabetic retinopathy: prevention and intervention studies. Prog Retin Eye Res.

[96] Kern TS, Engerman RL. Comparison of retinal lesions in alloxan-diabetic rats and galactose-fed rats. Curr Eye Res 1994, 1;13(12):863–7. 10.3109/02713689409015087 7720392

[97] Roy S, Lorenzi M (1996). Early biosynthetic changes in the diabetic-like retinopathy of galactose-fed rats. Diabetologia.

[98] Kobayashi T, Kubo E, Takahashi Y, Kasahara T, Yonezawa H, Akagi Y (1998). Retinal vessel changes in galactose-fed dogs. Arch Ophthalmol.

[99] Joussen AM, Doehmen S, Le ML, Koizumi K, Radetzky S, Krohne TU, Poulaki V, Semkova I, Kociok N (2009). TNF-α mediated apoptosis plays an important role in the development of early diabetic retinopathy and long-term histopathological alterations. Mol Vis.

[100] Kern TS, Engerman RL (1996). A mouse model of diabetic retinopathy. Arch Ophthalmol.

[101] Joussen AM, Poulaki V, Le ML, Koizumi K, Esser C, Janicki H, Schraermeyer U, Kociok N, Fauser S, Kirchhof B (2004). A central role for inflammation in the pathogenesis of diabetic retinopathy. FASEB J.

[102] Azrad-Leibovich T, Zahavi A, Gohas MF, Brookman M, Barinfeld O, Muhsinoglu O, Michowiz S, Fixler D, Goldenberg-Cohen N (2022). Characterization of Diabetic Retinopathy in two mouse models and response to a single injection of anti-vascular endothelial growth factor. Int J Mol Sci.

[103] Jiang X, Yang L, Luo Y (2015). Animal models of diabetic retinopathy. Curr Eye Res.

[104] Barber AJ, Antonetti DA, Kern TS, Reiter CE, Soans RS, Krady JK, Levison SW, Gardner TW, Bronson SK (2005). The Ins2Akita mouse as a model of early retinal complications in diabetes. Invest Ophthalmol Vis Sci.

[105] Bogdanov P, Corraliza L, Villena A, Carvalho J, Garcia-Arumi AR, Ramos J, Ruberte D, Simo J, Hernandez R (2014). The db/db mouse: a useful model for the study of diabetic retinal neurodegeneration. PLoS ONE.

[106] Rakoczy EP, Rahman ISA, Binz N, Li C-R, Vagaja NN, de Pinho M, Lai C-M (2010). Characterization of a mouse model of hyperglycemia and retinal neovascularization. Am J Pathol.

[107] Chaurasia SS, Lim RR, Parikh BH, Wey YS, Tun BB, Wong TY, Luu CD, Agrawal R, Ghosh A, Mortellaro A, Rackoczy E (2018). The NLRP3 inflammasome may contribute to pathologic neovascularization in the advanced stages of diabetic retinopathy. Sci Rep.

[108] Schmidt RE, Dorsey DA, Beaudet LN, Peterson RG (2003). Analysis of the Zucker Diabetic fatty (ZDF) type 2 diabetic rat model suggests a neurotrophic role for insulin/IGF-I in diabetic autonomic neuropathy. Am J Pathol.

[109] Schroeder M, Zagoory-Sharon O, Shbiro L, Marco A, Hyun J, Moran TH, Bi S, Weller A (2009). Development of obesity in the Otsuka Long-Evans Tokushima fatty rat. Am J Physiol Regul Integr Comp Physiol.

[110] Akimoto T, Nakama K, Katsuta Y, Zhang X-J, Ohsuga M, Ishizaki M, Sawai N, Ozawa H (2008). Characterization of a novel congenic strain of diabetic fatty (WBN/Kob-Leprfa) rat. Biochem Biophys Res Commun.

[111] Masuyama T, Komeda K, Hara A, Noda M, Shinohara M, Oikawa T, Kanazawa Y, Taniguchi K (2004). Chronological characterization of diabetes development in male spontaneously Diabetic Torii rats. Biochem Biophys Res Commun.

[112] Guest PC. Characterization of the Goto-Kakizaki (GK) rat model of type 2 diabetes. Methods Mol Biol 2019, 1916:203–11. 10.1007/978-1-4939-8994-2_19 30535697

[113] Izumi T, Yokota-Hashimoto H, Zhao S, Wang J, Halban PA, Takeuchi T (2003). Dominant negative pathogenesis by mutant proinsulin in the Akita diabetic mouse. Diabetes.

[114] Wang J, Takeuchi T, Tanaka S, Kubo S-K, Kayo T, Lu D, Takata K, Koizumi A, Izumi T (1999). A mutation in the insulin 2 gene induces diabetes with severe pancreatic β-cell dysfunction in the Mody mouse. J Clin Investig.

[115] Leiter EH. The NOD mouse: a model for analyzing the interplay between heredity and environment in development of autoimmune disease. ILAR J 1993, 35(1):4–14.

[116] Serreze DV, Chapman HD, Varnum DS, Gerling I, Leiter EH, Shultz LD (1997). Initiation of autoimmune diabetes in NOD/Lt mice is MHC class I-dependent. J Immunol.

[117] Pearson JA, Wong FS, Wen L (2016). The importance of the non obese Diabetic (NOD) mouse model in autoimmune diabetes. J Autoimmun.

[118] Thayer TC, Wilson SB, Mathews CE (2010). Use of nonobese diabetic mice to understand human type 1 diabetes. Endocrin Metab Clin.

[119] Chen H, Charlat O, Tartaglia LA, Woolf EA, Weng X, Ellis SJ, Lakey ND, Culpepper J, More KJ, Breitbart RE (1996). Evidence that the diabetes gene encodes the leptin receptor: identification of a mutation in the leptin receptor gene in db/db mice. Cell.

[120] Hummel KP, Dickie MM, Coleman DL (1966). Diabetes, a new mutation in the mouse. Science.

[121] Okamoto N, Tobe T, Hackett SF, Ozaki H, Vinores MA, LaRochelle W, Zack DJ, Campochiaro PA (1997). Transgenic mice with increased expression of vascular endothelial growth factor in the retina: a new model of intraretinal and subretinal neovascularization. Am J Pathol.

[122] Tee L, Penrose M, O’Shea J, Lai C-M, Rakoczy E, Dunlop S (2008). VEGF-induced choroidal damage in a murine model of retinal neovascularisation. Br J Ophthalmol.

[123] McLenachan S, Magno AL, Ramos D, Catita J, McMenamin PG, Chen FK, Rakoczy EP, Ruberte J (2015). Angiography reveals novel features of the retinal vasculature in healthy and diabetic mice. Exp Eye Res.

[124] Yokoi N, Hoshino M, Hidaka S, Yoshida E, Beppu M, Hoshikawa R, Sudo K, Kawada A, Takagi S, Seino S (2013). A novel rat model of type 2 diabetes: the Zucker fatty diabetes mellitus ZFDM rat. J Diabetes Res.

[125] Miyamoto K, Hiroshiba N, Tsujikawa A, Ogura Y. In vivo demonstration of increased leukocyte entrapment in retinal microcirculation of diabetic rats. Invest Ophthalmol Vis Sci. 1998;39(11):2190–4. 9761301

[126] Watanabe TK, Suzuki M, Yamasaki Y, Okuno S, Hishigaki H, Ono T, Oga K, Mizoguchi-Miyakita A, Tsuji A, Kanemoto N (2005). Mutated G‐protein‐coupled receptor GPR10 is responsible for the hyperphagia/dyslipidaemia/obesity locus of Dmo1 in the OLETF rat. Clin Exp Pharmacol Physiol.

[127] Lu ZY, Bhutto IA, Amemiya T (2003). Retinal changes in Otsuka long-evans Tokushima fatty rats (spontaneously diabetic rat)—possibility of a new experimental model for diabetic retinopathy. Jpn J Ophthalmol.

[128] Hornum L, Rømer J, Markholst H. The diabetes-prone BB rat carries a frameshift mutation in Ian4, a positional candidate of Iddm1. Diabetes 2002, 51(6):1972–9. 10.2337/diabetes.51.6.1972 12031988

[129] MacMurray AJ, Moralejo DH, Kwitek AE, Rutledge EA, Van Yserloo B, Gohlke P, Speros SJ, Snyder B, Schaefer J, Bieg S (2002). Lymphopenia in the BB rat model of type 1 diabetes is due to a mutation in a novel immune-associated nucleotide (Ian)-related gene. Genome Res.

[130] Rutledge EA, Fuller JM, Van Yserloo B, Moralejo DH, Ettinger RA, Gaur P, Hoehna JL, Peterson MR, Jensen R, Kwitek AE. Sequence variation and expression of the Gimap gene family in the BB rat. *Exp Diabetes Res* 2009, 2009. 10.1155/2009/835650 PMC2676327 19421422

[131] Miyamura N, Amemiya T (1998). Lens and retinal changes in the WBN/Kob rat (spontaneously diabetic strain). Ophthalmic Res.

[132] Mori M, Fu X, Chen L, Zhang G, Higuchi K (2009). Hereditary pancreatitis model WBN/Kob rat strain has a unique haplotype in the Pdwk1 region on chromosome 7. Exp Anim.

[133] Kakehashi A, Saito Y, Mori K, Sugi N, Ono R, Yamagami H, Shinohara M, Tamemoto H, Ishikawa SE, Kawakami M, Kanazawa Y (2006). Characteristics of diabetic retinopathy in SDT rats. Diabetes Metab Res Rev.

[134] Sasase T, Ohta T, Masuyama T, Yokoi N, Kakehashi A, Shinohara M (2013). The spontaneously diabetic torii rat: an animal model of nonobese type 2 diabetes with severe diabetic complications. J Diabetes Res.

[135] Shinohara M, Masuyama T, Shoda T, Takahashi T, Katsuda Y, Komeda K, Kuroki M, Kakehashi A, Kanazaw Y (2000). A new spontaneously diabetic non-obese Torii rat strain with severe ocular complications. Int J Exp Diabetes Res.

[136] Yamada H, Yamada E, Higuchi A, Matsumura M (2005). Retinal neovascularisation without ischaemia in the spontaneously diabetic Torii rat. Diabetologia.

[137] Movassat J, Calderari S, Fernández E, Martín MA, Escrivá F, Plachot C, Gangnerau MN, Serradas P, Alvarez C, Portha B (2007). Type 2 diabetes–a matter of failing β-cell neogenesis? Clues from the GK rat model. Diabetes Obes Metab.

[138] Calderari S, Gangnerau MN, Thibault M, Meile MJ, Kassis N, Alvarez C, Portha B, Serradas P (2007). Defective IGF2 and IGF1R protein production in embryonic pancreas precedes beta cell mass anomaly in the goto–kakizaki rat model of type 2 diabetes. Diabetologia.

[139] Miyamoto K, Ogura Y, Nishiwaki H, Matsuda N, Honda Y, Kato S, Ishida H, Seino Y (1996). Evaluation of retinal microcirculatory alterations in the Goto-Kakizaki rat. A spontaneous model of non-insulin-dependent diabetes. Invest Ophthalmol Vis Sci.

[140] Han Z, Guo J, Conley SM, Naash MI (2013). Retinal angiogenesis in the Ins2Akita mouse model of diabetic retinopathy. Invest Ophthalmol Vis Sci.

[141] Gastinger MJ, Kunselman AR, Conboy EE, Bronson SK, Barber AJ (2008). Dendrite remodeling and other abnormalities in the retinal ganglion cells of Ins2Akita diabetic mice. Invest Ophthalmol Vis Sci.

[142] Li CR, Sun SG (2010). VEGF expression and cell apoptosis in NOD mouse retina. Int J Ophthalmol.

[143] Zorrilla-Zubilete MA, Yeste A, Quintana FJ, Toiber D, Mostoslavsky R, Silberman DM (2018). Epigenetic control of early neurodegenerative events in diabetic retinopathy by the histone deacetylase SIRT 6. J Neurochem.

[144] Lee S, Harris NR. Losartan and ozagrel reverse retinal arteriolar constriction in non-obese diabetic mice. Microcirculation 2008, 15(5):379–87. 10.1080/10739680701829802 PMC2527598 18574741

[145] Shaw SG, Boden JP, Biecker E, Reichen J, Rothen B (2006). Endothelin antagonism prevents diabetic retinopathy in NOD mice: a potential role of the angiogenic factor adrenomedullin. Exp Biol Med.

[146] Clements RS, Robison WG, Cohen MP (1998). Anti-glycated albumin therapy ameliorates early retinal microvascular pathology in db/db mice. J Diabetes Complicat.

[147] Ding Y, Yuan S, Liu X, Mao P, Zhao C, Huang Q, Zhang R, Fang Y, Song Q, Yuan D, Xie P (2014). Protective effects of astragaloside IV on db/db mice with diabetic retinopathy. PLoS ONE.

[148] Cheung AK, Fung MK, Lo AC, Lam TT, So KF, Chung SS, Chung SK (2005). Aldose reductase deficiency prevents diabetes-induced blood-retinal barrier breakdown, apoptosis, and glial reactivation in the retina of db/db mice. Diabetes.

[149] Bogdanov P, Corraliza L, Villena A, Carvalho J, Garcia-Arumi AR, Ramos J, Ruberte D, Simo J, Hernandez R (2014). The db/db mouse: a useful model for the study of diabetic retinal neurodegeneration. PLoS ONE.

[150] Samuels IS, Bell BA, Pereira A, Saxon J, Peachey NS (2015). Early retinal pigment epithelium dysfunction is concomitant with hyperglycemia in mouse models of type 1 and type 2 diabetes. J Neurophysiol.

[151] van Eeden PE, Tee LB, Lukehurst S, Lai C-M, Rakoczy EP, Beazley LD, Dunlop SA (2006). Early vascular and neuronal changes in a VEGF transgenic mouse model of retinal neovascularization. Invest Ophthalmol Vis Sci.

[152] Shen W-Y, Lai C-M, Graham C, Binz N, Lai Y, Eade J, Guidolin D, Ribatti D, Dunlop S, Rakoczy P (2006). Long-term global retinal microvascular changes in a transgenic vascular endothelial growth factor mouse model. Diabetologia.

[153] Wisniewska-Kruk J, Klaassen I, Vogels IM, Magno AL, Lai C-M, Van Noorden CJ, Schlingemann RO, Rakoczy EP (2014). Molecular analysis of blood–retinal barrier loss in the Akimba mouse, a model of advanced diabetic retinopathy. Exp Eye Res.

[154] Danis RP, Yang Y (1993). Microvascular retinopathy in the Zucker diabetic fatty rat. Invest Ophthalmol Vis Sci.

[155] Kowluru RA, Mishra M, Kowluru A, Kumar B (2016). Hyperlipidemia and the development of diabetic retinopathy: comparison between type 1 and type 2 animal models. Metabolism.

[156] Szabó K, Énzsöly A, Dékány B, Szabó A, Hajdú RI, Radovits T, Mátyás C, Oláh A, Laurik LK, Somfai GM, Merkely B (2017). Histological evaluation of diabetic neurodegeneration in the retina of Zucker diabetic fatty (ZDF) rats. Sci Rep.

[157] Katsuda Y, Ohta T, Miyajima K, Kemmochi Y, Sasase T, Tong B, Shinohara M, Yamada T (2014). Diabetic complications in obese type 2 diabetic rat models. Exp Anim.

[158] Behl Y, Krothapalli P, Desta T, DiPiazza A, Roy S, Graves DT (2008). Diabetes-enhanced tumor necrosis factor-α production promotes apoptosis and the loss of retinal microvascular cells in type 1 and type 2 models of diabetic retinopathy. Am J Pathol.

[159] Wohlfart P, Lin J, Dietrich N, Kannt A, Elvert R, Herling AW, Hammes HP (2014). Expression patterning reveals retinal inflammation as a minor factor in experimental retinopathy of ZDF rats. Acta Diabetol.

[160] Bhutto IA, Lu Z-Y, Takami Y, Amemiya T (2002). Retinal and choroidal vasculature in rats with spontaneous diabetes type 2 treated with the angiotensin-converting enzyme inhibitor cilazapril: corrosion cast and electron-microscopic study. Ophthalmic Res.

[161] Shirao Y, Kawasaki K (1998). Electrical responses from diabetic retina. Prog Retin Eye Res.

[162] Matsuura T, Yamagishi S, Kodama Y, Shibata R, Ueda S, Narama I (2005). Otsuka Long-Evans Tokushima fatty (OLETF) rat is not a suitable animal model for the study of angiopathic diabetic retinopathy. Int J Tissue React.

[163] Sima AA, Bouchier M, Christensen H (1983). Axonal atrophy in sensory nerves of the diabetic BB-Wistar rat: a possible early correlate of human diabetic neuropathy. Ann Neurol.

[164] Sima AA, Chakrabarti S, Garcia-Salinas R, Basu PK (1985). The BB-rat-an authentic model of human diabetic retinopathy. Curr Eye Res.

[165] Robinson WG, McCaleb ML, Feld LG, Michaelis OE, And NL, Mercandetti M (1991). Degenerated intramural pericytes (‘ghost cells’) in the retinal capillaries of diabetic rats. Curr Eye Res.

[166] Blair NP, Tso MO, Dodge JT (1984). Pathologic studies of the blood–retinal barrier in the spontaneously diabetic BB rat. Invest Ophthalmol Vis Sci.

[167] Miyamura N, Amemiya T (1998). Lens and retinal changes in the WBN/Kob rat (spontaneously Diabetic strain) Electron-microscopic study. Ophthal Res.

[168] Tsuji N, Matsuura T, Ozaki K, Sano T, Narama I (2009). Diabetic retinopathy and choroidal angiopathy in diabetic rats (WBN/Kob). Exp Anim.

[169] Matsuura T, Horikiri K, Ozaki K, Narama I (1999). Proliferative retinal changes in diabetic rats (WBN/Kob). Comp Med.

[170] Ogawa T, Ohira A, Amemiya T (1998). Superoxide dismutases in retinal degeneration of WBN/Kob rat. Curr Eye Res.

[171] Fukuda M, Nakanishi Y, Fuse M, Yokoi N, Hamada Y, Fukagawa M, Negi A, Nakamura M (2010). Altered expression of aquaporins 1 and 4 coincides with neurodegenerative events in retinas of spontaneously diabetic Torii rats. Exp Eye Res.

[172] Matsuoka M, Ogata N, Minamino K, Matsumura M (2007). Leukostasis and pigment epithelium-derived factor in rat models of diabetic retinopathy. Mol Vis.

[173] Sasase T, Morinaga H, Abe T, Miyajima K, Ohta T, Shinohara M, Matsushita M, Kakehashi A (2009). Protein kinase C beta inhibitor prevents diabetic peripheral neuropathy, but not histopathological abnormalities of retina in spontaneously Diabetic Torii rat. Diabetes Obes Metab.

[174] Carmo A, Cunha-Vaz J, Carvalho A, Lopes M (2000). Nitric oxide synthase activity in retinas from non-insulin-dependent diabetic Goto-Kakizaki rats: correlation with blood–retinal barrier permeability. Nitric Oxide.

[175] Yatoh S, Mizutani M, Yokoo T, Kozawa T, Sone H, Toyoshima H, Suzuki S, Shimano H, Kawakami Y, Okuda Y, Yamada N (2006). Antioxidants and an inhibitor of advanced glycation ameliorate death of retinal microvascular cells in diabetic retinopathy. Diabetes Metab Res Rev.

[176] Berdugo M, Delaunay K, Lebon C, Naud MC, Radet L, Zennaro L, Picard E, Daruich A, Beltrand J, Kermorvant-Duchemin E, Polak M (2021). Long-term oral treatment with non-hypoglycemic dose of glibenclamide reduces diabetic retinopathy damage in the goto-kakizaki rat model. Pharmaceutics.

[177] Gong CY, Lu B, Sheng YC, Yu ZY, Zhou JY, Ji LL (2016). The development of diabetic retinopathy in Goto-Kakizaki rat and the expression of angiogenesis-related signals. Chin J Physiol.

[178] Hachana S, Pouliot M, Couture R, Vaucher E (2020). Diabetes-induced inflammation and vascular alterations in the goto–kakizaki rat retina. Curr Eye Res.

[179] Kim MK, Kim SG, Lee SK (2020). 4-Hexylresorcinol-induced angiogenesis potential in human endothelial cells. Maxillofac Plast Reconstr Surg.

[180] Durham JT, Dulmovits BM, Cronk SM, Sheets AR, Herman IM (2015). Pericyte chemomechanics and the angiogenic switch: insights into the pathogenesis of proliferative diabetic retinopathy?. Invest Ophthalmol Vis Sci.

[181] Gong Y, Fu Z, Edin ML, Liu C-H, Wang Z, Shao Z, Fredrick TW, Saba NJ, Morss PC, Burnim SB (2016). Cytochrome P450 oxidase 2 C inhibition adds to ω-3 long-chain polyunsaturated fatty acids protection against retinal and choroidal neovascularization. Atertio Thromb Vasc Biol.

[182] Yu Z, Zhang T, Gong C, Sheng Y, Lu B, Zhou L, Ji L, Wang Z. Erianin inhibits high glucose-induced retinal angiogenesis via blocking ERK1/2-regulated HIF-1α-VEGF/VEGFR2 signaling pathway. Sci Rep 2016, 6(1):1–15. 10.1038/srep34306 PMC5039671 27678303

[183] Johnen S, Djalali-Talab Y, Kazanskaya O, Möller T, Harmening N, Kropp M, Izsvák Z, Walter P, Thumann G (2015). Antiangiogenic and neurogenic activities of sleeping Beauty-mediated PEDF-transfected RPE cells in vitro and in vivo. BioMed Res Int.

[184] Spuul P, Daubon T, Pitter B, Alonso F, Fremaux I, Kramer I, Montanez E, Génot E (2016). VEGF-A/Notch-induced podosomes proteolyse basement membrane collagen-IV during retinal sprouting angiogenesis. Cell Rep.

[185] Siemerink MJ, Hughes MR, Dallinga MG, Gora T, Cait J, Vogels IM, Yetin-Arik B, Van Noorden CJ, Klaassen I, McNagny KM (2016). CD34 promotes pathological epi-retinal neovascularization in a mouse model of oxygen-induced retinopathy. PLoS ONE.

[186] Bergers G, Song S (2005). The role of pericytes in blood-vessel formation and maintenance. Neuro Oncol.

[187] Yanni SE, Clark ML, Yang R, Bingaman DP, Penn JS (2010). The effects of nepafenac and amfenac on retinal angiogenesis. Brain Res Bull.

[188] Moleiro A, Conceição G, Leite-Moreira A, Rocha-Sousa A. A critical analysis of the available in vitro and ex vivo methods to study retinal angiogenesis. J Ophthalmol 2017, 2017. 10.1155/2017/3034953 PMC5564124 28848677

[189] Rezzola S, Belleri M, Ribatti D, Costagliola C, Presta M, Semeraro F (2013). A novel ex vivo murine retina angiogenesis (EMRA) assay. Exp Eye Res.

[190] Schnichels S, Paquet-Durand F, Löscher M, Tsai T, Hurst J, Joachim SC, Klettner A (2021). Retina in a dish: cell cultures, retinal explants and animal models for common diseases of the retina. Prog Retin Eye Res.

[191] Lüke M, Januschowski K, Lüke J, Peters S, Wirtz N, Yörük E, Lüke C, Bartz-Schmidt KU, Grisanti S, Szurman P (2009). The effects of ranibizumab (Lucentis) on retinal function in isolated perfused vertebrate retina. Br J Ophthalmol.

[192] Forrester JV, Chapman A, Kerr C, Roberts J, Lee WR, Lackie JM (1990). Bovine retinal explants cultured in collagen gels: a model system for the study of proliferative retinopathy. Arch Ophthalmol.

[193] Nowak-Sliwinska P, Alitalo K, Allen E, Anisimov A, Aplin AC, Auerbach R, Augustin HG, Bates DO, van Beijnum JR, Bender RH, Bergers G (2018). Consensus guidelines for the use and interpretation of angiogenesis assays. Angiogenesis.

[194] Alarautalahti V, Ragauskas S, Hakkarainen JJ, Uusitalo-Järvinen H, Uusitalo H, Hyttinen J, Kalesnykas G, Nymark S (2019). Viability of mouse retinal explant cultures assessed by preservation of functionality and morphology. Invest Ophthalmol Vis Sci.

[195] Murakami T, Suzuma K, Takagi H, Kita M, Ohashi H, Watanabe D, Ojima T, Kurimoto M, Kimura T, Sakamoto A (2006). Time-lapse imaging of vitreoretinal angiogenesis originating from both quiescent and mature vessels in a novel ex vivo system. Invest Ophthalmol Vis Sci.

[196] Amato R, Biagioni M, Cammalleri M, Dal Monte M, Casini G (2016). VEGF as a survival factor in ex vivo models of early diabetic retinopathy. Invest Ophthalmol Vis Sci.

[197] Chantelau E, Meyer-Schwickerath R, Klabe K. Downregulation of serum IGF-1 for treatment of early worsening of diabetic retinopathy: a long-term follow-up of two cases. Ophthalmol 2010, 224(4):243–6. 10.1159/000260231 19940532

[198] Haurigot V, Villacampa P, Ribera A, Bosch A, Ramos D, Ruberte J, Bosch F (2012). Long-term retinal PEDF overexpression prevents neovascularization in a murine adult model of retinopathy. PLoS ONE.

[199] Simo R, Carrasco E, Garcia-Ramirez M, Hernandez C (2006). Angiogenic and antiangiogenic factors in proliferative diabetic retinopathy. Curr Diabetes Rev.

[200] Whitmire W, Al-Gayyar MM, Abdelsaid M, Yousufzai BK, El-Remessy AB (2011). Alteration of growth factors and neuronal death in diabetic retinopathy: what we have learned so far. Mol Vis.

[201] Sawamiphak S, Ritter M, Acker-Palmer A (2010). Preparation of retinal explant cultures to study ex vivo tip endothelial cell responses. Nat Protoc.

[202] Sadikan MZ, Abdul Nasir NA. Diabetic retinopathy: emerging concepts of current and potential therapy. Naunyn Schmiedebergs Arch Pharmacol. 2023:1-12.10.1007/s00210-023-02599-y 10.1007/s00210-023-02599-y 37401966

[203] Sadikan MZ, Nasir NAA, Ghani NAA, Lambuk L, Iezhitsa IN, Agarwal R. The Use of Fiji Image J as an Image Analysis Tool for Measuring Retinal Vessel Diameter in Rodent Model of Diabetic Retinopathy. Asian J Med Biomed. 2021;5(1):61-6. 10.37231/ajmb.2021.5.1.422

[204] Abdul Ghani NA, Abdul Nasir NA, Lambuk L, Sadikan MZ, Agarwal R, Ramli N. The effect of palm oil-derived tocotrienol-rich fraction in preserving normal retinal vascular diameter in streptozotocin-induced diabetic rats. Graefes Arch Clin Exp Ophthalmol. 2023;261(6):1587-96. 10.1007/s00417-022-05965-3 10.1007/s00417-022-05965-3 36622408

[205] Sadikan MZ, Abdul Nasir NA, Agarwal R, Mohd Ismail N. Protective effect of palm oil-derived tocotrienol-rich fraction against retinal neurodegenerative changes in rats with streptozotocin-induced diabetic retinopathy. Biomolecules 2020;10(4):556. 10.3390/biom10040556 10.3390/biom10040556 PMC7226502 32260544

